# Combination of subtherapeutic anti-TNF dose with dasatinib restores clinical and molecular arthritogenic profiles better than standard anti-TNF treatment

**DOI:** 10.1186/s12967-021-02764-y

**Published:** 2021-04-23

**Authors:** Lydia Ntari, Christoforos Nikolaou, Ksanthi Kranidioti, Dimitra Papadopoulou, Eleni Christodoulou-Vafeiadou, Panagiotis Chouvardas, Florian Meier, Christina Geka, Maria C. Denis, Niki Karagianni, George Kollias

**Affiliations:** 1 Biomedcode Hellas SA, Vari, Greece; 2 Institute for Bioinnovation, Biomedical Sciences Research Center (BSRC), Alexander Fleming, 34 Alexander Fleming Street, 16672 Vari, Greece; 3 Department of Medical Oncology, Inselspital, University Hospital and University of Bern, Bern, Switzerland; 4 Department for BioMedical Research (DBMR), University of Bern, Bern, Switzerland; 5 Division of Rheumatology, University Hospital Frankfurt, Goethe University, Frankfurt am Main, Germany; 6 Fraunhofer Institute for Molecular Biology and Applied Ecology IME, Project Group Translational Medicine and Pharmacology TMP, Frankfurt am Main, Germany; 7 Department of Physiology and Joint Rheumatology Program, School of Medicine, National and Kapodistrian University of Athens, Athens, Greece

**Keywords:** Arthritis, Tyrosine Kinase inhibitors, Dasatinib, Bosutinib, Tofacitinib, Anti-hTNF, Combination therapy, Chronic inflammation

## Abstract

**Background:**

New medications for Rheumatoid Arthritis (RA) have emerged in the last decades, including Disease Modifying Antirheumatic Drugs (DMARDs) and biologics. However, there is no known cure, since a significant proportion of patients remain or become non-responders to current therapies. The development of new mode-of-action treatment schemes involving combination therapies could prove successful for the treatment of a greater number of RA patients.

**Methods:**

We investigated the effect of the Tyrosine Kinase inhibitors (TKIs) dasatinib and bosutinib, on the human TNF-dependent Tg197 arthritis mouse model. The inhibitors were administered either as a monotherapy or in combination with a subtherapeutic dose of anti-hTNF biologics and their therapeutic effect was assessed clinically, histopathologically as well as via gene expression analysis and was compared to that of an efficient TNF monotherapy.

**Results:**

Dasatinib and, to a lesser extent, bosutinib inhibited the production of TNF and proinflammatory chemokines from arthritogenic synovial fibroblasts. Dasatinib, but not bosutinib, also ameliorated significantly and in a dose-dependent manner both the clinical and histopathological signs of Tg197 arthritis. Combination of dasatinib with a subtherapeutic dose of anti-hTNF biologic agents, resulted in a synergistic inhibitory effect abolishing all arthritis symptoms. Gene expression analysis of whole joint tissue of Tg197 mice revealed that the combination of dasatinib with a low subtherapeutic dose of Infliximab most efficiently restores the pathogenic gene expression profile to that of the healthy state compared to either treatment administered as a monotherapy.

**Conclusion:**

Our findings show that dasatinib exhibits a therapeutic effect in TNF-driven arthritis and can act in synergy with a subtherapeutic anti-hTNF dose to effectively treat the clinical and histopathological signs of the pathology. The combination of dasatinib and anti-hTNF exhibits a distinct mode of action in restoring the arthritogenic gene signature to that of a healthy profile. Potential clinical applications of combination therapies with kinase inhibitors and anti-TNF agents may provide an interesting alternative to high-dose anti-hTNF monotherapy and increase the number of patients responding to treatment.

**Supplementary Information:**

The online version contains supplementary material available at 10.1186/s12967-021-02764-y.

## Background

Rheumatoid Arthritis (RA) is a common chronic inflammatory disease estimated to affect approximately 1% of the worldwide population and is a leading cause of disability. It affects primarily the joints manifesting initially with signs of synovial inflammation, eventually progressing to cartilage and bone destruction, while it can also affect other organs, including eyes, lungs and heart [1].

While there is no known cure for RA, nonetheless medications have emerged in the last two decades slowing disease progression and preventing joint deformity, thus dramatically improving the long-term management of RA [2]. Medications currently available in the clinic include Non-steroidal anti-inflammatory drugs (NSAIDs), corticosteroids, disease-modifying anti-rheumatic drugs (DMARDs) as well as biological response modifiers [3]. Despite the therapeutic potential of such medications however, a subset of RA patients may not tolerate or may be resistant to certain treatments, while the issue of treatment-related side effects, including the higher risk of infections due to immunosuppression, remain important complications [4]. To address such issues, combination therapies have become an attractive therapeutic approach, as combining drugs targeting different pathogenic pathways has shown improved effectiveness compared to monotherapies [5].

Kinases play a central role in cellular and cytokine responses related to RA pathogenesis and have thus recently emerged as attractive alternative therapeutic targets for the treatment of RA [6]. In line with this, small-molecule tyrosine kinase inhibitors (TKIs), such as the JAK inhibitors tofacitinib, baricitinib and upadacitinib have recently been approved for the treatment of patients with moderate-to-severe active RA, who had an inadequate response or intolerance to at least one DMARD [7, 8], while several other second-generation JAK inhibitors are also currently under development and in clinical trials [9]. Additional TKIs are also emerging as RA therapeutics, with fostamatinib showing promising results in treating RA pathology [10] and imatinib and nilotinib showing effectiveness in ameliorating signs of pathology in mouse models of arthritis [11, 12]. Dasatinib and bosutinib, two second-generation TKIs have been shown to display anti-inflammatory activities [13], sharing targets with imatinib and nilotinib [14], while also exhibiting their own target specificities [15].

In the present study, we sought to assess the therapeutic potential of dasatinib and bosutinib, in the human TNF-driven Tg197 RA mouse model [16], administered as a monotherapy but also, and in accordance with the clinical practice of administering DMARDs in combination with biologics for the treatment of RA patients, in combination with anti-hTNF biologics. Our work shows that dasatinib, but not bosutinib, has a significant therapeutic effect as a monotherapy, but, more importantly, it is mostly effective when administered in combination with subtherapeutic doses of anti-hTNF inhibitors.

## Materials and methods

### Mice and treatments

WT and human TNF transgenic mice (Tg197) [16] were bred and maintained in a mixed CBA × C57BL/6 J genetic background in the animal facilities of Biomedcode Hellas S.A. under specific pathogen-free conditions. Animals were housed in standard plastic cages with wood chip bedding, under an inverted 12:12-h light/dark cycle at a constant temperature of 22 ± 2 °C and relative humidity of approximately 60%. Standard diet and water were provided ad libitum. Experiments were approved by the BSRC Al. Fleming Institutional Committee of Protocol Evaluation in conjunction with the Veterinary Service Management of the Hellenic Republic Prefecture of Attica according to all current European and national legislation and were performed in accordance with relevant guidelines and regulations. For efficacy drug evaluation experiments, which were reproduced at least twice, mice were distributed in gender-balanced groups of 8 animals, 4 from each gender. Drug administration started at the 3rd week of age and lasted 6–7 weeks with the following dosing schemes and routes of administration: Infliximab (Janssen), adalimumab (AbbVie) and golimumab (Janssen) were administered intraperitoneally twice weekly; etanercept (Pfizer) was administered subcutaneously twice or thrice weekly; dasatinib, bosutinib (LC Labs, USA) as well as tofacitinib (Selleckchem) were administered twice daily by oral gavage. Daily doses were calculated on a dose-per-kilogram (mg/kg) basis for each individual mouse. Dasatinib and bosutinib were dissolved in DMSO and further diluted in 50% propylene glycol (Chemco) [17], which was also used as vehicle. Tofacitinib was dissolved in 0.5% methylcellulose/0.025% Tween 20 (Sigma-Aldrich, USA) [18], which was also used as vehicle. Mice were monitored regularly and weight and arthritic scores were recorded on a weekly basis as previously described [19].

### Histology

Joint tissues were fixed in 4% aqueous formaldehyde solution, demineralized in 13% EDTA/0.1 M sodium phosphate and paraffin embedded in the sagittal plane. Sections were stained with H&E, TRAPs (tartrate-resistant acid phosphatase staining) and toluidine blue. Histopathological evaluation was performed blindly according to a previously described scoring system [19], in which synovitis, bone erosion and cartilage destruction are assessed either altogether (0–4 scale) or individually (0–3 scale). Paraffin-embedded longitudinal heart sections were also stained with H&E staining. Images were acquired with Leica DM2500 microscope equipped with Leica SFL4000 camera (Leica Microsystems).

### Synovial fibroblast isolation and cytokine detection

Primary mouse SFs were isolated as previously described [20] from at least 3 Tg197 animals at their 8th week of age. Each drug treatment was tested in technical triplicates. The levels of hTNF, mouse CCL5/RANTES and CCL20/MIP-3 were assessed in the supernatants of SFs after a 48 h incubation with the indicated treatments. hTNF was measured using Quantikine HS immunoassays (R&D Systems, Minneapolis, Minnesota, USA) according to manufacturer’s protocol. CCL5/RANTES and CCL20/MIP-3 levels were measured using mouse DuoSet ELISA, (R&D Systems, Minneapolis, Minnesota, USA), according to the manufacturer’s instructions.

### Statistical analysis

Statistical analysis of in vitro and in vivo data was performed using two-tailed Mann–Whitney test or unpaired *t*-test.

### RNA preparation, gene expression profiling and analysis

Total RNA was isolated from whole hind limb joint tissues of 3 mice from each experimental treatment group, using Trizol® reagent in combination with the RNeasy mini kit (Qiagen), according to the manufacturer’s guidelines. All samples were hybridized on the Affymetrix GeneChip Mouse Gene 2.0 ST array. Data analysis, identification of differentially expressed genes, comparison and clustering of differential expression profiles and assessment of treatment efficiency at transcriptional level were performed as previously described [21]. Normalized gene expression values as well as differential expression values (logFC) for the complete set of analyzed genes are provided in Additional files 2 and 3, respectively (Mendeley Data: 10.17632/vnzbpxx7c2.1).

## Results

### Dasatinib suppresses the arthritogenic phenotype of Tg197-derived SFs more efficiently than bosutinib

**Fig. 1:**
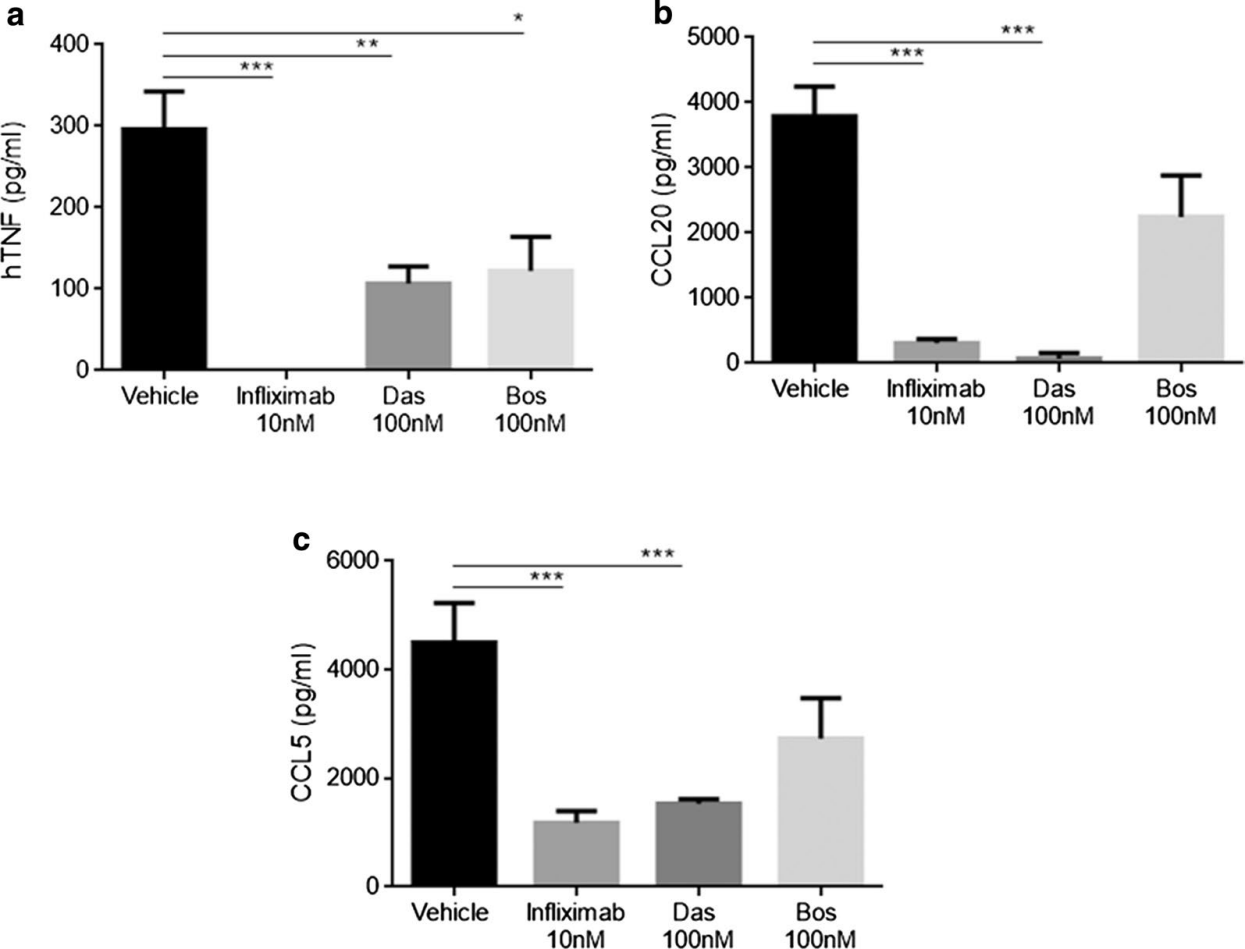
Treatment with dasatinib reduces SF-secreted levels of inflammatory proteins in vitro. Tg197 synovial fibroblast-secreted hTNF (**a**), CCL20 (**b**), CCL5 (**c**) levels following a 48 h treatment with Infliximab (10 nM), dasatinib (100 nM) or bosutinib (100 nM). (* p-value < 0.05; ** p-value < 0.01; *** p-value ≤ 0.0001). All data are shown as mean ± SEM and all comparisons were made against vehicle

We investigated the effect of dasatinib and bosutinib on synovial fibroblasts, the key cellular orchestrators of both human [22, 23] and Tg197 [24] RA pathogenesis. Dasatinib- and bosutinib-treated SFs, isolated from 8-week-old Tg197 mice with established RA pathology [16, 25], exhibited greatly reduced levels of secreted hTNF (Fig. 1a). Furthermore, dasatinib, and, to a lesser degree, bosutinib greatly reduced the levels of CCL5 and CCL20, two chemokines that have been associated with synovial activation [26, 27], reaching levels of inhibition similar to the ones observed with Infliximab treatment (Fig. 1b, c). These data indicate that while both inhibitors affect the activated phenotype of Tg197-derived SFs, dasatinib appears to be superior to bosutinib.

### Dasatinib, but not bosutinib, effectively attenuates Tg197 RA severity in a dose dependent manner

**Fig. 2:**
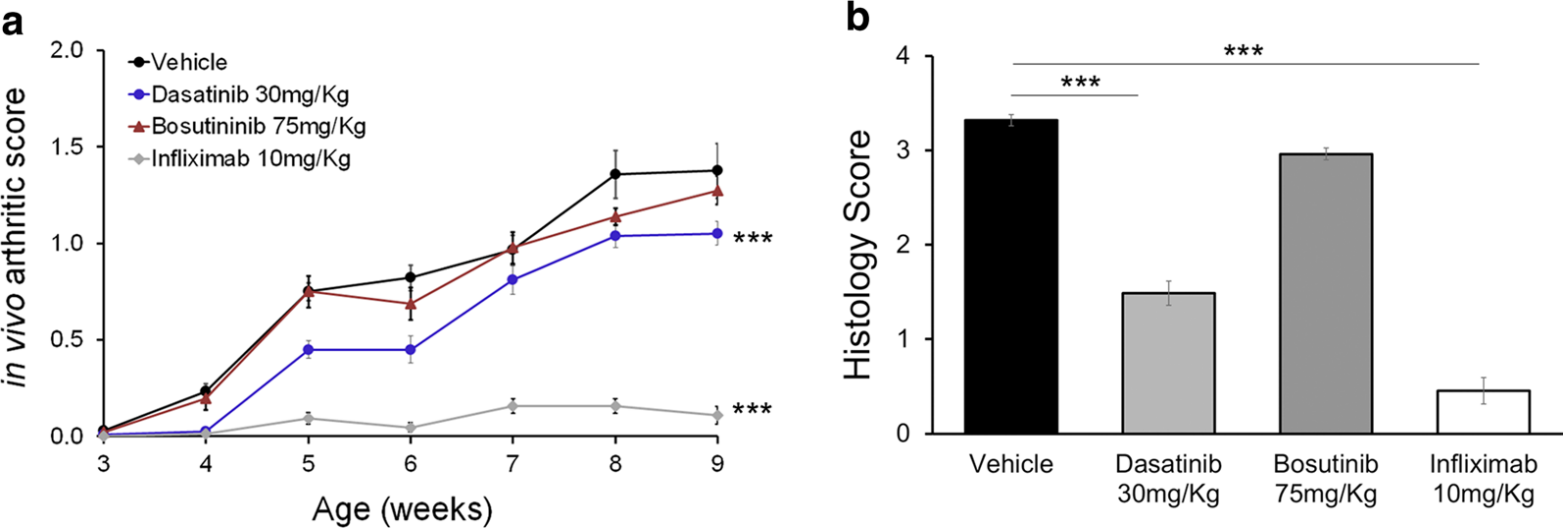
Dasatinib, and not bosutinib, treatment ameliorates arthritis symptoms. Clinical (**a**) and histopathological (**b**) arthritis scores showing the response of Tg197 animals treated with dasatinib (30 mg/Kg) or bosutinib (75 mg/Kg) in comparison to Infliximab (10 mg/Kg) treatment. (** p-value < 0.01; *** p-value ≤ 0.0001). All data are shown as mean ± SEM and all comparisons were made against vehicle

Given the suppressive effect of dasatinib and bosutinib on Tg197-derived SFs, we also studied their in vivo effect on Tg197 arthritis pathology. Dasatinib and bosutinib were administered at doses of 30 mg/Kg and 75 mg/Kg respectively, doses previously shown to be effective in other mouse models of human pathologies [17, 28], and they were compared to an effective therapeutic dose of 10 mg/Kg Infliximab (Additional file 1; Figure S1). Dasatinib significantly ameliorated both the clinical and histopathological signs of Tg197 arthritic pathology (Fig. 2a, b), albeit less efficiently than Infliximab. Bosutinib on the other hand, did not exhibit any therapeutic effect in ameliorating neither the clinical nor the histopathological parameters of Tg197 arthritic pathology (Fig. 2a, b), further highlighting the therapeutic advantage of dasatinib.

**Fig. 3:**
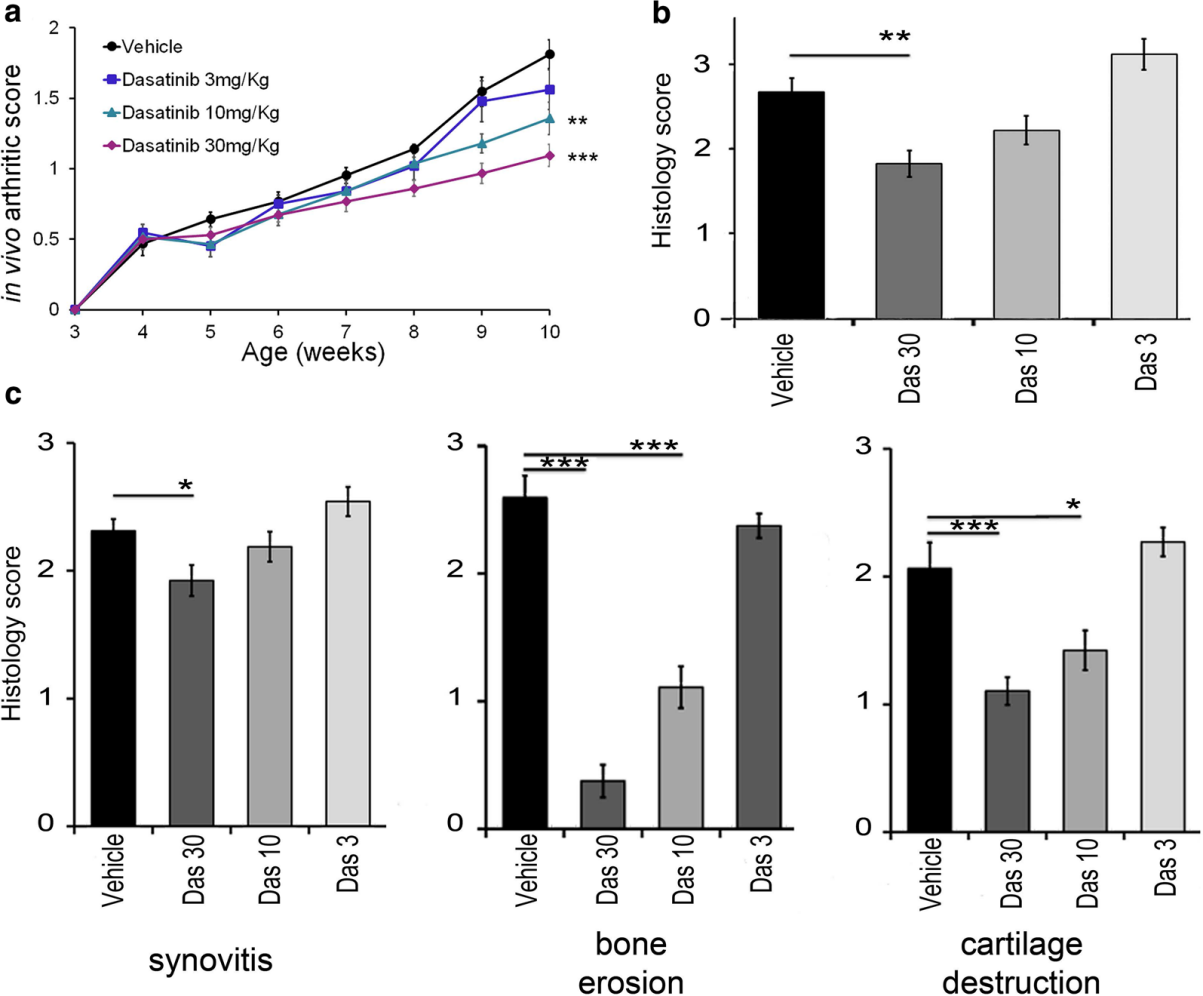
Dose-dependent amelioration of arthritis symptoms following treatment with dasatinib. Clinical (**a**)**,** histopathological (**b**), as well as individual histopathological arthritis (**c**) scores depicting the dose-dependent response of Tg197 animals to dasatinib treatment. (* p-value < 0.05; ** p-value < 0.01; *** p-value ≤ 0.0001). All data are shown as mean ± SEM and all comparisons were made against vehicle

Furthermore, dasatinib exhibited a dose dependent therapeutic effect, with the doses of 30 and 10 mg/Kg significantly reducing the clinical RA severity (Fig. 3a), while overall histopathological signs of arthritis were significantly inhibited only by the 30 mg/Kg dose (Fig. 3b). Interestingly, a more detailed examination of the effect of dasatinib on specific features of the arthritic pathology revealed that the 10 mg/Kg dose of dasatinib significantly reduced bone erosion and cartilage destruction, while the highest dose of 30 mg/Kg dasatinib significantly ameliorated all three features, including synovitis (Fig. 3c). The dose dependency of the therapeutic effect of dasatinib on the arthritic pathology supports the specificity of its inhibitory effect, which however remains suboptimally effective since it cannot fully abolish it.

### Treatment with a combination of dasatinib and a subtherapeutic dose of Infliximab completely abolishes the Tg197 arthritis pathology

Given that combination therapies with DMARDs are recommended for the management of RA and its related comorbidities [29], we explored the possibility of enhancing the therapeutic effect of dasatinib on both arthritis and RA-related valvular pathology developed in Tg197 mice [30], by combining it with a low, 1 mg/Kg, suboptimal dose of the anti-hTNF biologic Infliximab (Additional file 1; Figure S1).

**Fig. 4:**
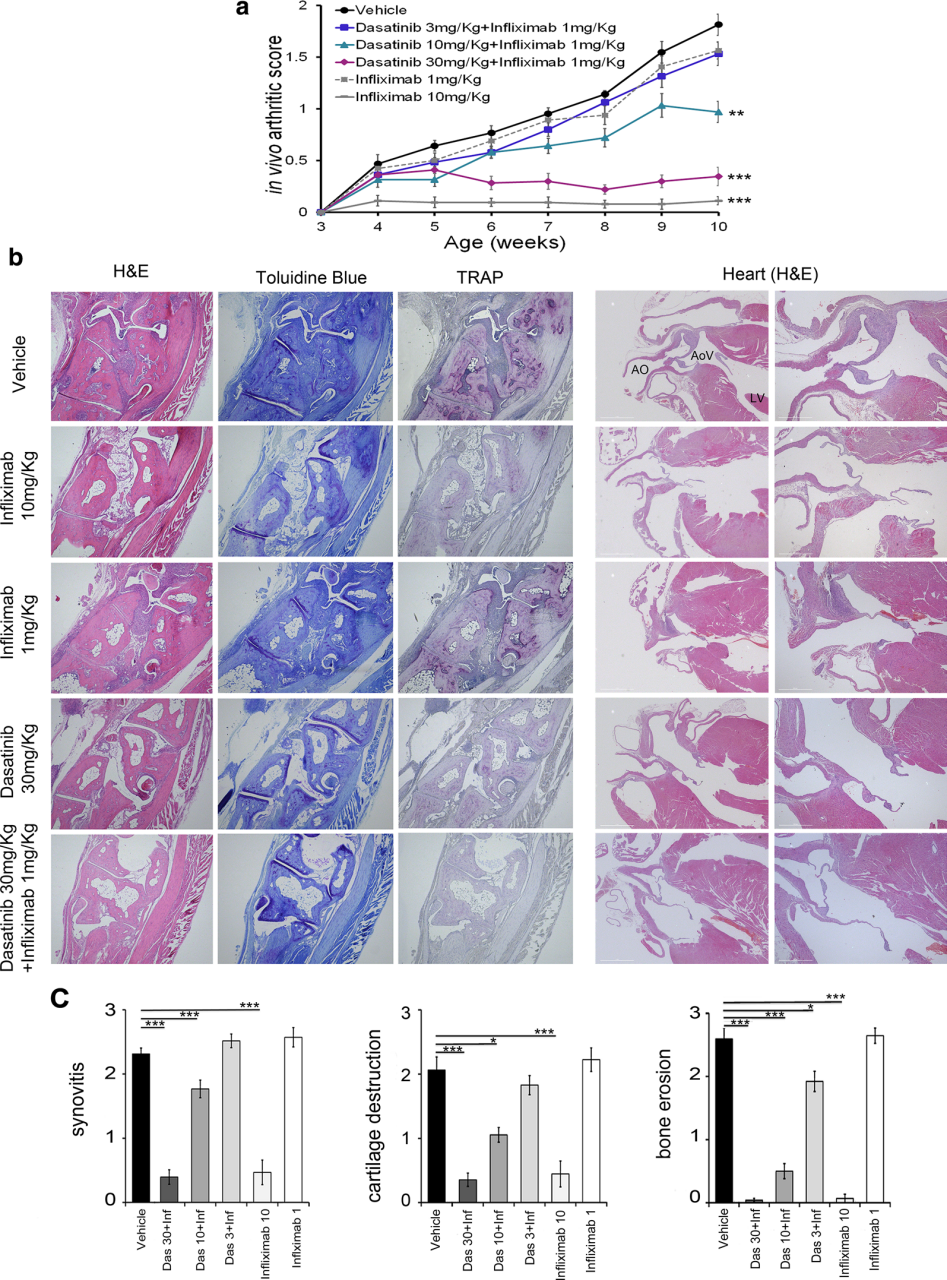
Combination of dasatinib and a subtherapeutic dose of Infliximab completely abolishes arthritis pathology and its related valvular pathology. **a** Clinical arthritis score depicting the synergistic therapeutic effect of a combination of different doses of dasatinib with 1 mg/Kg of Infliximab, in comparison to two doses of Infliximab (** p-value < 0.01; *** p-value ≤ 0.0001). All data are shown as mean ± SEM and all comparisons were made against vehicle. **b** Representative histology images depicting the amelioration of Tg197 arthritis symptoms and valvular pathology following treatment with dasatinib alone or in combination with a low dose of Infliximab. Hind limb paraffin sections are stained with H&E for the evaluation of synovitis, toluidine blue for the evaluation of cartilage destruction and TRAP for the evaluation of osteoclast numbers and bone erosion. Longitudinal heart paraffin sections are stained with H&E for the evaluation of the valvular pathology. [Right panel scale bar: 500 μm, left panel scale bar: 1 mm (AO: Aorta, AoV: Aortic valve, LV: left ventricle)]. **c** Individual histopathological arthritis scores depicting the synergistic therapeutic effect of a combination of different doses of dasatinib with 1 mg/Kg of Infliximab, in comparison to two doses of Infliximab (* p-value < 0.05; *** p-value ≤ 0.0001). All data are shown as mean ± SEM and all comparisons were made against vehicle

Interestingly, the therapeutic effects of the 3,10 or 30 mg/Kg doses of dasatinib, were greatly enhanced when combined with a dose of 1 mg/Kg Infliximab both at the clinical (Fig. 4a) and the histopathological level (Fig. 4b, c). More specifically, the 30 mg/Kg dose of dasatinib combined with 1 mg/Kg Infliximab achieved an inhibition of the clinical and histopathological signs of arthritis similar to that achieved with the high therapeutic dose of 10 mg/Kg Infliximab monotherapy (Fig. 4a, b). Additionally, the combination therapy of 30 mg/Kg dasatinib and 1 mg/Kg Infliximab ameliorated the histopathological signs of valvular pathology that develops in these mice, which are the extensive valvular thickening and fibrosis of the aortic and mitral valves, to a similar extent to that achieved from high therapeutic dose of 10 mg/Kg Infliximab monotherapy (Fig. 4b).

A more detailed examination of specific histopathological features of arthritis, revealed that combination therapy with 10 or 30 mg/Kg dasatinib significantly ameliorated synovitis, bone erosion and cartilage destruction (Fig. 4b, c), while combination therapy with 3 mg/Kg dasatinib also showed an enhanced inhibitory effect that significantly reduced bone erosion (Fig. 4b, c). Again, the combination of 30 mg/Kg dasatinib and 1 mg/Kg Infliximab ameliorated all three pathology features to an extent similar to the one observed with the high therapeutic dose of Infliximab (Fig. 4b, c).

**Fig. 5:**
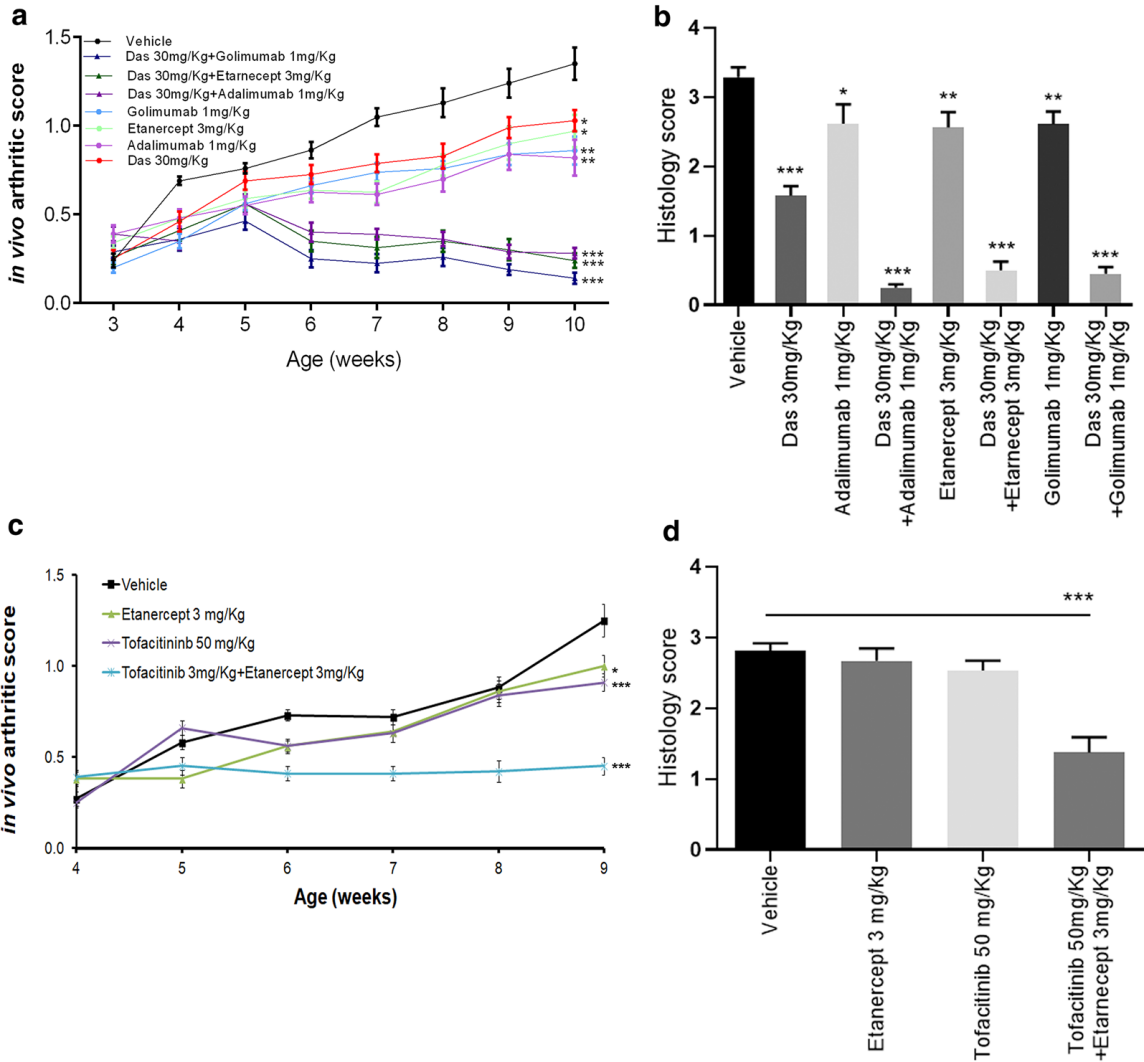
Combination of dasatinib and tofacitinib with a subtherapeutic dose of anti-hTNF agents ameliorate arthritis pathology. Clinical (**a**) and histopathological (**b**) arthritis scores showing the response of Tg197 animals treated with dasatinib 30 mg/Kg alone or in combination with 3 mg/Kg Etanercept (twice weekly) or 1 mg/Kg Adalimumab, Infliximab or Golimumab (twice weekly) (* p-value < 0.05; **p-value; < 0.01***p-value ≤ 0.0001, comparisons made to vehicle). All data are shown as mean ± SEM. Clinical (**c**) and histopathological (**d**) arthritis scores showing the response of Tg197 animals treated with 3 mg/Kg Etanercept (thrice weekly) alone, tofacitinib 50 mg/Kg alone or in combination of the two drugs (* p-value < 0.05; ***p-value ≤ 0.0001, comparisons made to vehicle). All data are shown as mean ± SEM

Overall, the therapeutic effect of the combination therapy on both arthritis and heart valve pathology was superior to that achieved with dasatinib and low dose Infliximab monotherapies, thus suggesting not simply an additive but rather a synergistic effect of the two drugs (Fig. 4). This synergistic effect was dasatinib-specific, since combination of bosutinib with a suboptimal dose of Infliximab did not have a similar effect on arthritis pathology (Additional file 1; Figure S2). We further investigated the possible synergistic effect of 30 mg/Kg dasatinib in combination with additional anti-TNF agents, that in high-dose monotherapies ameliorate arthritis (Additional file 1; Figure S3). All anti-TNF biologics tested at subtherapeutic doses, including the monoclonal antibodies, adalimumab and golimumab as well as the receptor fusion protein etanercept, exhibited similar enhanced therapeutic activity when combined with 30 mg/Kg dasatinib (Fig. 5a, b). Moreover, we tested the possible synergistic effect of an ineffective dose of tofacitinib, a JAK inhibitor with known subtherapeutic effect on RA [31], with the anti-TNF agent etanercept. Interestingly, this combination therapy significantly ameliorated both clinical and histopathological signs of arthritis, thus indicating a potential for a broader range of kinase inhibitors to synergize with anti-TNF agents to efficiently treat arthritis pathology (Fig. 5c, d).

### Transcriptome analysis reveals a unique drug-specific signature and synergistic superiority of the dasatinib-Infliximab combination treatment

**Fig. 6:**
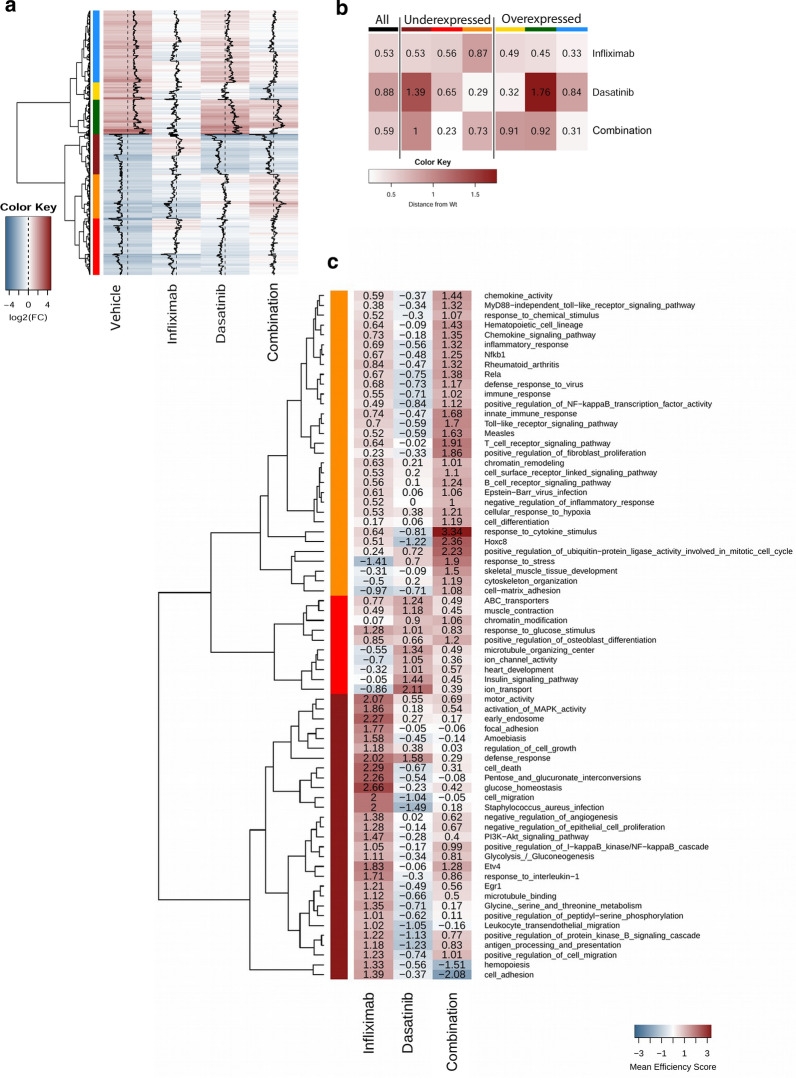
Treatment of Tg197 animals with dasatinib alone or in combination with a low dose of Infliximab effectively restores gene expression to normal levels. **a** Differential gene expression levels against WT samples for 867 disease-associated differentially expressed genes. The colored bars on the left side of the heatmap correspond to 6 gene clusters on the basis of their expression pattern across all conditions. Clustering was performed with agglomerative hierarchical clustering using Ward's objective function maximization. Dashed vertical lines represent WT baseline levels, while black full lines correspond to differential expression levels (red: over-expression, blue: under-expression). **b** Mean gene expression distance from WT for all 867 genes (black) and the six differentially expressed clusters. Cluster characterization is identical with that shown in (**a**). Distance is measured as the mean absolute logFC value against WT controls for each cluster. Small values correspond to greater similarities to the healthy condition*.* **c** Treatment efficiency scores taking into account differential expression levels against both diseased (Tg197) and WT controls. Genes that were differentially expressed against either WT or diseased (Tg197) animals in any of the three treatments, or between diseased and healthy controls, were selected and then mapped to GO terms and KEGG pathways. For each functional category, an efficiency score was calculated as the log10 of the ratio of mean logFC against disease over mean logFC against WT controls (see Methods for details). High scores indicate effective recovery of gene expression to normal levels without disrupting the healthy state. Only functions with an efficiency score > 1 for at least one of the treatments are shown. The colored bars on the left side of the heatmap correspond to 3 clusters of functional terms created on the basis of efficiency scores across all conditions

To gain insights in the differential modes of action of dasatinib and Infliximab monotherapies as well as of the combination therapy in the treatment of arthritis, we analyzed the expression profiles of whole joints of Tg197 mice treated with these treatments and compared them for a set of 867 Tg197 disease-associated genes, previously defined by the comparison of expression profiles of Tg197- with WT-derived whole joints [21] (Additional file 2) (Fig. 6). All three treatments studied, i.e. 30 mg/Kg dasatinib monotherapy, 10 mg/Kg Infliximab monotherapy and the combination of 30 mg/Kg dasatinib with 1 mg/Kg Infliximab, affected this set of disease-associated genes, albeit in distinct ways which allowed us to distinguish the effects of the different treatments. More specifically, Infliximab monotherapy effectively restored the expression of overexpressed genes to WT levels (upper part of Fig. 6a), affecting however to a lesser extent the set of underexpressed genes (lower part of the heatmap, Fig 6a). Dasatinib monotherapy, on the other hand, exhibited an opposite profile restoring more efficiently the underexpressed rather than the overexpressed set of genes to WT levels, though exhibiting an overall inferior therapeutic profile to that of Infliximab (Fig. 6a). Notably, the combination therapy restored the gene expression of a large proportion of both over- and underexpressed genes to WT levels, thus suggesting a potential treatment scheme for functional modulation of the arthritic phenotype (Fig. 6a).

The therapeutic potential of the different treatments was further assessed quantitatively by measuring the aggregate gene expression changes (Fig. 6b). Mean expression distance analysis of the 867 disease-related genes, revealed that the combination treatment has a similar effect to the one observed with the 10 mg/Kg Infliximab monotherapy (Fig. 6b). However, when looking at the two largest hierarchical individual gene clusters (red, blue) that represent approximately 50% of the total differentially expressed genes, we observe that the combination therapy is more efficient in restoring these clusters to the WT condition (closer to 0), compared to the average of either dasatinib or Infliximab monotherapies (Fig. 6b). This overall enhanced efficiency that exceeds the positive effect of either of the two drugs, strongly suggests a synergistic effect of the combination treatment.

Gene expression changes following treatment may not be confined specifically to disease-associated genes [21] and in fact, large numbers of genes not belonging to the 867 Tg197 disease-associated gene signature, exhibited significantly altered expression patterns following the different treatments (Additional file 1; Figure S4). In order to assess differential expression in a broader scale and at a functional level, we performed functional enrichment analysis on an extended set of 3733 altered genes, which included all genes being differentially expressed either in diseased mice or in any one of the three treatments (Fig. 6c). We then went on to calculate the aggregate efficiency scores for all functional categories (GO, KEGG pathways and TF targets) by comparing gene expression distances from both WT and diseased samples, as previously described [21] (Fig. 6c). A high aggregate efficiency score in this type of analysis indicates that the treatment can efficiently restore the Tg197 pathogenic profile to resemble that of the WT, while negative values indicate a significant deviation from the normal, WT, profile.

Focusing our analysis on all the functional categories that have an aggregate efficiency score higher that 1 in at least one of the treatments, we identified three distinct clusters, one specific for each treatment (Fig. 6c). The 10 mg/Kg Infliximab monotherapy exhibited an expected anti-inflammatory effect, indicated by the restoration of genes related to rheumatoid arthritis, chemokine activity, (innate) immune response, B and T cells, as well as TLRs activation, inflammatory response and NF-κB activation pathways. In addition, Infliximab monotherapy restored to the WT condition, genes related to SF activation, as indicated by its effect on pathways such as fibroblast proliferation, cell migration, cell growth, cell differentiation and cytoskeleton organization. Dasatinib monotherapy on the other hand specifically restored functions involved in osteoblast activation, in accordance with its previously reported effect on osteoclast/osteoblast physiology [32]. It also exhibited, however, a significant number of pathways with negative efficiency scores suggesting its inability to efficiently restore the diseased profile. Most interestingly, the combination therapy was found to restore, with increased efficiency, the same inflammation-related pathways restored by the Infliximab monotherapy, thus suggesting underlying synergistic effects in the combined treatment, while the number of negative efficiency scores was low suggesting a better efficiency in restoring the arthritogenic phenotype. Interestingly, combination therapy can also restore pathways that are minimally affected by Infliximab monotherapy, such as cytoskeleton organization and cell–matrix adhesion, both of which are associated with the arthritogenic phenotype of Tg197 SFs [33, 34]. Overall, the combined administration of Infliximab and dasatinib achieves comparable or higher efficiency scores across the full spectrum of the examined pathways.

## Discussion

The introduction of new disease modifying and disease-controlling biologics has improved the quality of life of RA patients by allowing better management of pain and disease progression. Nonetheless, there are still unmet needs in the treatment of RA, as a significant subset of patients remain resistant to existing therapies or may become non-responders during their treatment [3] and may also experience serious side effects [35]. Therefore, it is important to develop new therapeutics and new therapeutic approaches that will effectively cover these unmet needs. Drug repositioning as well as combination therapies have become attractive approaches towards this direction.

Dasatinib and bosutinib are two second-generation TKIs with known anti-inflammatory activities [13], that are currently used for the treatment of imatinib-resistant CML and Philadelphia chromosome-positive acute lymphoblastic leukemia [36] and have also been shown to have an effect on inflammatory pathologies, [37–40]. They both inhibit a broad range of kinases, sharing some of their targets with the first-generation TKIs imatinib and nilotinib, that have shown effectiveness in mouse models of arthritis [11, 12]. They also, however, have additional specific targets [41] some of which could be targets for the treatment of RA [42, 43]. In line with this, dasatinib has been recently reported to be effective in ameliorating the pathology of the Collagen Induced Arthritis (CIA) [44].

To further study the potential repositioning of dasatinib and bosutinib for the treatment of arthritis, we took advantage of the hTNFTg mice (Tg197), which spontaneously develop human TNF-driven arthritis pathology [16], similar to that observed in a subset of human RA patients responsive to anti-hTNF treatment [45]. In Tg197 mice, synovial fibroblasts (SFs) play a key pathogenic role [46], similar to the human RA pathology and we show here that both TKIs have a suppressive effect on parameters of the activated arthritogenic phenotype of Tg197-derived SFs. These data suggest that these two TKIs may have the potential to interrupt the persistent pathogenic crosstalk between synovial stromal cells and immune cells thus slowing down the progression of the arthritis pathology. A similar ability of dasatinib to control the arthritogenic phenotype of human RASFs has been previously reported [44].

When tested in vivo, dasatinib reduced synovial inflammation, cartilage destruction and bone erosion, while bosutinib was ineffective in controlling any of the in vivo or histopathological signs of arthritis. This difference of the two TKIs could be attributed to their differential mode of action [47–49]. Among their differences, it is worth mentioning that dasatinib, has the potential to target KIT and platelet-derived growth factor receptor (PDGFR) [50], as well as collagen receptor tyrosine kinases discoidin domain receptors 1 and 2 (DDR1, 2) [51], PI3K and ERK, potential targets for the treatment of RA [52, 53].

The most marked effect of dasatinib was observed in its ability to protect from cartilage destruction and bone erosion. These findings are in line with previous reports supporting that dasatinib demonstrates convergent bone anabolic [32] and reduced bone resorption effects [32], osteoclast inhibitory functions [54], enhancement of chondrogenesis [55] as well as enhanced bone preservation in metastatic cancers [56, 57]. Moreover, a similar effect has been recently reported for the effect of dasatinib in the CIA model of arthritis [44]. These findings, together with our data, support that dasatinib has the potential to moderate bone-related pathologies in arthritis. Since dasatinib has also been proven efficacious in ameliorating the pathology of CIA [44], it is possible that the benefits of the synergistic effect of dasatinib and a low dose of anti-hTNF therapy may also prove beneficial in CIA, as well as in other arthritis models [58].

As dasatinib monotherapy proved only partially effective in the Tg197 model of arthritis, we tested its combination with a subtherapeutic dose of Infliximab, a widely used anti-hTNF agent. Interestingly, this combination treatment efficiently abolished both the in vivo clinical and histopathological signs of arthritis, as well as the comorbid heart valve pathology developing in these mice [30], with an efficiency similar to that of a high dose of Infliximab. The therapeutic effect of the combination treatment exceeded by far the sum of the effects of either of the two components of the combination, i.e. dasatinib alone and a subtherapeutic dose of Infliximab alone, thus suggesting a synergistic function of these drugs. Characteristically, even the combination of the non-efficacious doses of 3 mg/Kg dasatinib and 1 mg/Kg anti-hTNF resulted in statistically significant amelioration of bone erosion, indicating that a low dose of anti-hTNF can greatly enhance the bone modification functions of even the lower doses of dasatinib [32].

Aiming at a better definition and understanding of the mode of action of the combination therapy, we made use of a recently published transcriptomic analysis framework and the respective gene expression and function-based profiles of the TNF-dependent arthritis pathology [21]. Mean expression distance analysis of the arthritic gene signature confirmed that the combination therapy had a similar therapeutic efficacy to the high dose anti-hTNF monotherapy, albeit exhibiting differential mechanisms, as the two treatments restored efficiently the expression of different gene clusters, with the combination therapy resulting in greatly improved responses for a more extensive set of genes. This higher efficiency of the combination therapy can be attributed to the modulation of the expression of separate sets of RA-related genes targeted by dasatinib and anti-hTNF, however this might not be sufficient to fully explain the synergy exhibited by their combination. As treatments may also affect the expression of genes beyond the disease profile, we performed functional analysis of a more extended set of genes including those deregulated following each of the treatments. This analysis revealed a striking potential of the combination therapy to positively modulate disease-related pathways and functions, achieving efficiencies higher than Infliximab monotherapy, including efficient restoration of pathways minimally affected by Infliximab monotherapy. As such pathways are critical in other RA models as well [58], it is possible that the effect of dasatinib, and more importantly, the synergistic effect of dasatinib and anti-TNF may play a crucial role in different mouse models and consequently in different subsets of RA patients. In fact, these results hold promise for the use of combination therapies to more efficiently treat RA patients that do not respond to monotherapies or conventional therapeutic schemes. It would be of interest to further explore the novel functional profile of the combination therapy we provide here to discover new biomarkers to predict the responsiveness of RA patients to dasatinib monotherapy or to its combination with subtherapeutic doses of anti-hTNF. A validation of such biomarkers discovered initially in pre-clinical studies, using RA mouse models, in human patients could prove both a cost-effective and useful tool to more efficiently treat specific subsets of RA patients.

Combination therapies that could be tested in the clinic may not necessarily need to be limited to dasatinib, as we provide evidence that other small molecule kinase inhibitors, such as tofacitinib, can also show a synergistic effect when combined with subtherapeutic doses of anti-hTNF treatment. As tofacitinib is an already approved drug for RA treatment, the therapeutic effect of its combination with anti-hTNF agents could be more easily evaluated in patients.

Overall, we provide here proof-of-principle experimental evidence that the synergistic therapeutic effect of the combination of dasatinib and anti-hTNF make it a viable therapeutic approach to be tested in the clinic for the treatment of RA. Reducing the dose of anti-hTNF may be beneficial in alleviating the side effects arising from this treatment [35], however, as the use of dasatinib, as is the case with other kinase inhibitors, has been also linked with increased risk of toxicities [59, 60], it is crucial to explore optimal dosing schemes in the clinic. We suggest that the development of new mode of action treatment schemes, involving combination therapies of dasatinib or other kinase inhibitors and low anti-TNF biologics, could prove beneficial in the clinic, provided that the necessary precautions related to their drug-drug interaction and toxicities are properly addressed.

## Conclusion

Overall, our findings support that combination of a subtherapeutic dose of Infliximab, a well-known anti-hTNF agent, with dasatinib, a known small molecule tyrosine kinase inhibitor, shows a strong therapeutic effect in TNF-driven arthritis and we show that, when combined, the two treatments act synergistically to restore, not only clinical, but also molecular arthritogenic profiles, better than standard anti-TNF treatment or treatment with dasatinib alone. These data highlight the potential therapeutic advantage of an alternative therapeutic scheme involving the combination of a low dose anti-TNF treatment with small molecule kinase inhibitors with potential clinical benefits for the treatment of arthritis and related comorbidities. Achieving the balance between optimal efficiency and minimal adverse events would contribute in establishing combination treatments as next generation therapies for the treatment of RA.

## Supplementary Information

**Additional file 1: Figure S1. ** Historical clinical (A) and histopathological (B) arthritic scores depicting the dose-dependent effect of Infliximab on Tg197 arthritic pathology. All data are shown as mean± SEM. **Figure S2.** Clinical (A) and histopathological (B) arthritic scores showing the response of Tg197 animals treated with bosutinib (75 mg/Kg) administered either alone or in combination with 1mg/Kg Infliximab in comparison to two doses of Infliximab (10mg/Kg). All data are shown as mean± SEM (***p-value≤0.0001). **Figure S3.** Historical clinical (A) and histopathological (B) arthritic scores showing the response of Tg197 animals to treatment with different commercially available anti-hTNF treatments (Infliximab 10mg/Kg, Adalimumab 10mg/Kg, Golimumab 3mg/Kg, and Etanercept 10mg/Kg). Infliximab, Adalimumab and Golimumab were administered intraperitoneally twice weekly. Etanercept was administered subcutaneously thrice weekly. All data are shown as mean± SEM. **Figure S4.** Set overlaps for 3733 genes whose expression was significantly altered (|logFC|>=1, adjusted p value<=0.05) compared to WT controls in at least one of the three treatments. Linked dots correspond to genes shared between categories, shown in the horizontal bars. Vertical bars correspond to the number of genes belonging to each set.

**Additional file 2.** Log-fold change and adjusted p-values for all genes using Wild-type condition as control.

**Additional file 3.** Normalized gene expression values for all genes in all replicates.

## Data Availability

All data that support the findings of this study are included in the article or uploaded as Additional files 1, 2, 3.

## Abbreviations

CCL: CC chemokine ligands
CIA: Collagen induced arthritis
CML: Chronic myelogenous leukemia
DDR: Discoidin domain receptors
DMARD: Disease modifying antirheumatic drugs
DMSO: Dimethyl sulfoxide
EDTA: Ethylenediaminetetraacetic acid
ERK: Extracellular signal regulated kinase
GO: Gene Ontology
H&E: Hematoxylin Eosin
hTNF: Human TNF
JAK: Janus kinase inhibitors
KEGG: Kyoto Encyclopedia of Genes and Genomes
Kg: Kilogram
mg: Milligram
MIP-3: Monocyte chemoattractant protein-3
NF-κB: Nuclear factor kappa-light-chain-enhancer of activated B cells
NSAIDs: Non-steroidal anti-inflammatory drugs
PDGFR: Platelet-derived growth factor receptor
PI3K: Phosphatidylinositol-3-kinase
RA: Rheumatoid arthritis
RANTES: Regulated on activation, normal T cell expressed and secreted
RNA: Ribonucleic acid
SFs: Synovial fibroblasts
TF: Transcription Factor
Tg: Transgenic
TKI: Tyrosine kinase inhibitor
TLRs: Toll-like receptors
TNF: Tumor Necrosis Factor
TRAP: Tartrate-resistant acid phosphatase
WT: Wild Type
