# Supplementary material for: Combination of subtherapeutic anti-TNF dose with dasatinib restores clinical and molecular arthritogenic profiles better than standard anti-TNF treatment
Source: J Transl Med. 2021 Apr 23;19:165. doi: 10.1186/s12967-021-02764-y (PMC8063445; doi:10.1186/s12967-021-02764-y)
Supplement: Supplementary file 1 — Additional file 1: Figure S1. Historical clinical (A) and histopathological (B) arthritic scores depicting the dose-dependent effect of Infliximab on Tg197 arthritic pathology. All data are shown as mean± SEM. Figure S2. Clinical (A) and histopathological (B) arthritic scores showing the response of Tg197 animals treated with bosutinib (75 mg/Kg) administered either alone or in combination with 1mg/Kg Infliximab in comparison to two doses of Infliximab (10mg/Kg). All data are shown as mean± SEM (***p-value≤0.0001). Figure S3. Historical clinical (A) and histopathological (B) arthritic scores showing the response of Tg197 animals to treatment with different commercially available anti-hTNF treatments (Infliximab 10mg/Kg, Adalimumab 10mg/Kg, Golimumab 3mg/Kg, and Etanercept 10mg/Kg). Infliximab, Adalimumab and Golimumab were administered intraperitoneally twice weekly. Etanercept was administered subcutaneously thrice weekly. All data are shown as mean± SEM. Figure S4. Set overlaps for 3733 genes whose expression was significantly altered (|logFC|>=1, adjusted p value<=0.05) compared to WT controls in at least one of the three treatments. Linked dots correspond to genes shared between categories, shown in the horizontal bars. Vertical bars correspond to the number of genes belonging to each set. [file 12967_2021_2764_MOESM1_ESM.docx]

**­Combination of subtherapeutic anti-TNF dose with dasatinib restores clinical and molecular arthritogenic profiles better than standard anti-TNF treatment.**

**Additional File 1**

**Supplementary figures**

Lydia Ntari^1^, Christoforos Nikolaou^2^, Ksanthi Kranidioti^1^, Dimitra Papadopoulou^2^, Eleni Christodoulou-Vafeiadou^1^, Panagiotis Chouvardas^3,4^, Florian Meier^5,6^, Christina Geka^1^, Maria C. Denis^1^, Niki Karagianni^1^ and George Kollias^2, 7*^

^1^ Biomedcode Hellas SA, Vari, Greece

^2^ Institute for Bioinnovation, Biomedical Sciences Research Center Alexander Fleming, Vari, Greece

^3^ Department of Medical Oncology, Inselspital, University Hospital and University of Bern, Switzerland

^4^ Department for BioMedical Research (DBMR), University of Bern, Bern, Switzerland

^5^ Division of Rheumatology, University Hospital Frankfurt, Goethe University, Frankfurt am Main, Germany

^6^ Fraunhofer Institute for Molecular Biology and Applied Ecology IME, Project Group Translational Medicine and Pharmacology TMP, Frankfurt am Main, Germany

^7^ Department of Physiology and Joint Rheumatology Program, School of Medicine, National and Kapodistrian University of Athens, Greece

*Corresponding Author

**Correspondence to**:

Professor George Kollias, Institute for Bioinnovation, Biomedical Sciences Research Center (BSRC), 'Alexander Fleming', 34 Alexander Fleming street, Vari, 16672 Greece

(contact details: [kollias@fleming.gr](mailto:kollias@fleming.gr), tel. +30-2109655310)

**
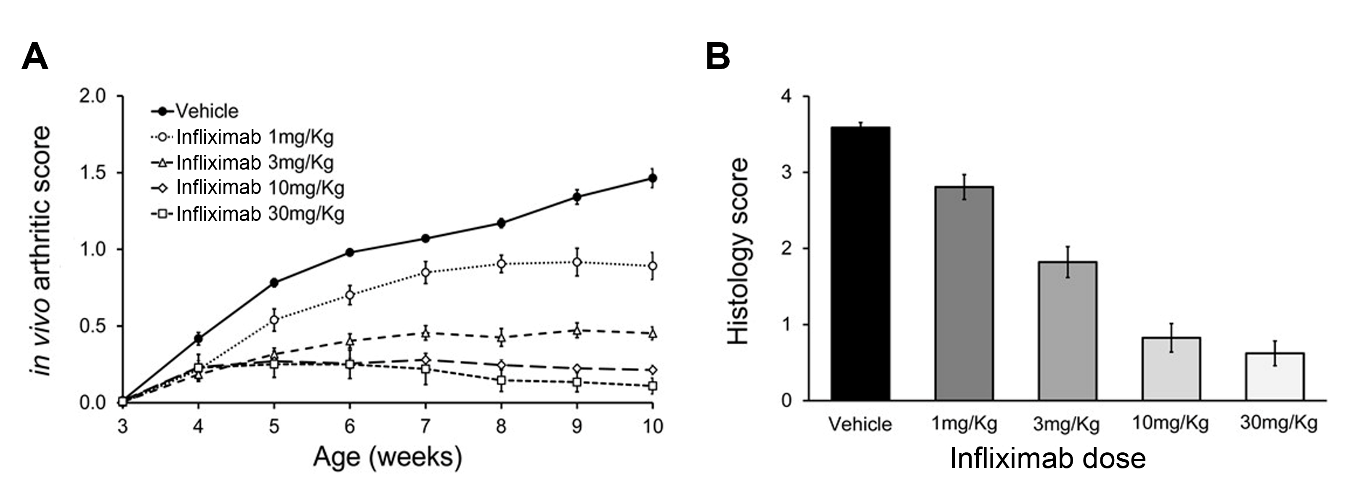
**

**Figure S1**: Historical clinical **(A)** and histopathological **(B)** arthritic scores depicting the dose-dependent effect of Infliximab on Tg197 arthritic pathology. All data are shown as mean± SEM.





**Figure S2:** Clinical **(A)** and histopathological **(B)** arthritic scores showing the response of Tg197 animals treated with bosutinib (75 mg/Kg) administered either alone or in combination with 1mg/Kg Infliximab in comparison to two doses of Infliximab (10mg/Kg). All data are shown as mean± SEM (***p-value≤0.0001).

**
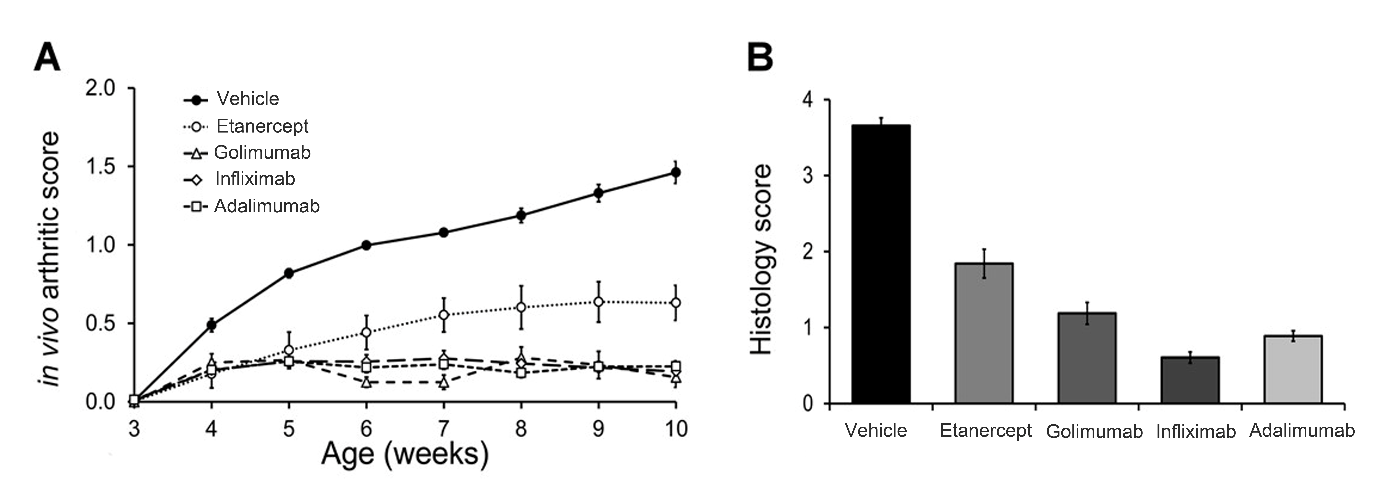
**

**Figure S3**: Historical clinical **(A)** and histopathological **(B)** arthritic scores showing the response of Tg197 animals to treatment with different commercially available anti-hTNF treatments (Infliximab 10mg/Kg, Adalimumab 10mg/Kg, Golimumab 3mg/Kg, and Etanercept 10mg/Kg). Infliximab, Adalimumab and Golimumab were administered intraperitoneally twice weekly. Etanercept was administered subcutaneously thrice weekly. All data are shown as mean± SEM.

**
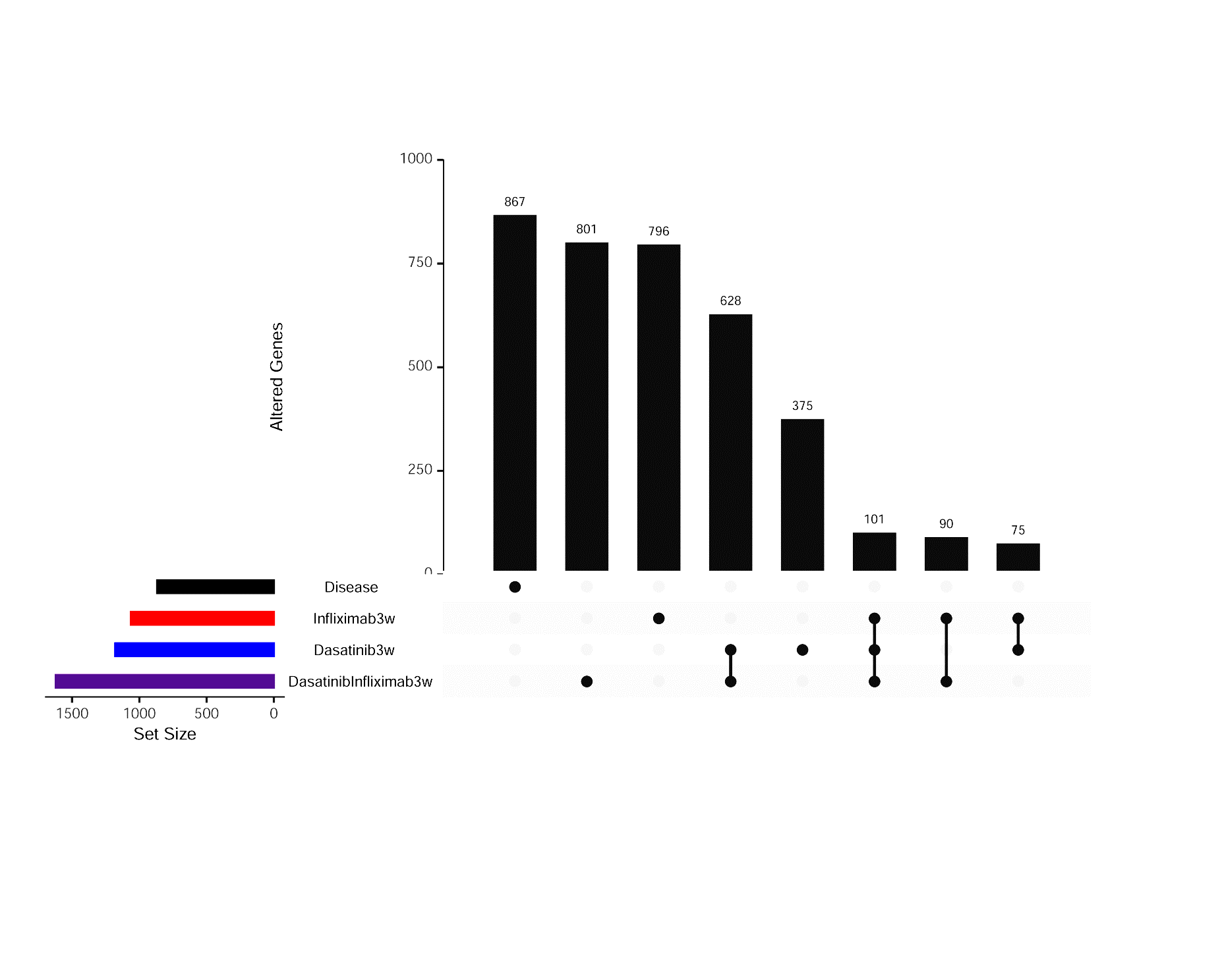
**

**Figure S4:** Set overlaps for 3733 genes whose expression was significantly altered (|logFC|>=1, adjusted p value<=0.05) compared to WT controls in at least one of the three treatments. Linked dots correspond to genes shared between categories, shown in the horizontal bars. Vertical bars correspond to the number of genes belonging to each set.
